# Expanded and updated data and a query pipeline for iBeetle-Base

**DOI:** 10.1093/nar/gkx984

**Published:** 2017-10-24

**Authors:** Jürgen Dönitz, Lizzy Gerischer, Stefan Hahnke, Stefan Pfeiffer, Gregor Bucher

**Affiliations:** Dpt. of Evolutionary Developmental Genetics, Georg August University of Göttingen, 37077 Göttingen, Germany; Institute of Bioinformatics, University Medical Center Göttingen (UMG) Georg August University, 37077 Göttingen, Germany; Institute for Mathematics and Computer Science, Ernst Moritz Arndt University, 17487 Greifswald, Germany

## Abstract

The iBeetle-Base provides access to sequence and phenotype information for genes of the beetle Tribolium castaneum. It has been updated including more and updated data and new functions. RNAi phenotypes are now available for >50% of the genes, which represents an expansion of 60% compared to the previous version. Gene sequence information has been updated based on the new official gene set OGS3 and covers all genes. Interoperability with FlyBase has been enhanced: First, gene information pages of homologous genes are interlinked between both databases. Second, some steps of a new query pipeline allow transforming gene lists from either species into lists with related gene IDs, names or GO terms. This facilitates the comparative analysis of gene functions between fly and beetle. The backend of the pipeline is implemented as endpoints of a RESTful interface, such that it can be reused by other projects or tools. A novel online interface allows the community to propose GO terms for their gene of interest expanding the range of animals where GO terms are defined. iBeetle-Base is available at http://ibeetle-base.uni-goettingen.de/

## INTRODUCTION

With the advent of next generation sequencing (NGS) the number of species with a sequenced genome or transcriptome is rapidly increasing and comprehensive inventories of genes become available for more and more taxa. However, very little is known about the functions of these genes outside the classical model systems. Conservation of sequence between orthologs often correlates with similar molecular functions but both subtle and profound differences in biological function have been observed frequently between for instance beetles and flies (1–3). In order to obtain a more balanced view on the function and evolution of genes, a comprehensive assessment of gene functions outside established model systems is needed. The red flour beetle *Tribolium castaneum* has been established as additional insect model organism. The genome assembly is quite good and was accepted as reference genome by NCBI (GCA_000002335.3). Besides an extended transgenic and genetic toolkit, *T. castaneum* shows a robust and systemic RNAi (4,5). Based on this, a genome-wide RNAi screen has been performed, the iBeetle screen (6). Phenotypic data for approximately 30% of the genes was gathered during this project and was published at iBeetle-Base along with respective gene sequence information. Since the first release of the database, iBeetle-Base was mined for various projects (7–12).

However, such a resource needs to aim for genome-wide coverage in order to provide maximum benefit for the community. Therefore, the data of a second screening phase was added to cover >50% of the genome and updated sequence information is now available genome-wide. Many genomics approaches result in long lists of genes involved in a process, which need further scrutiny. To match this need, a new query pipeline was implemented that allows transforming lists of gene IDs of either fly or beetle into other IDs and allows retrieving additional information like gene names and GO terms. Finally, we aim at allowing community contributions to iBeetle-Base. As a first step, an online page was implemented that gives the community the possibility to propose GO terms for the genes they study.

## RESULTS

### Expansion of the data by 60% and updated gene information

The initial dataset of iBeetle-Base was the result of the first phase of the unbiased large scale RNAi screen iBeetle performed in the red flour beetle *T. castaneum* (6,7). In that screening phase, around one third of the known *Tribolium* genes were knocked down, analysed and the phenotypic annotations published in the iBeetle-Base (7). Based on the recently finished second screening phase this data was extended by 3200 genes (60% increase) to cover ∼50% of the genome. Table 1 compares the updated content with the previous published version. The homology assignments for this release have been calculated by OrthoDB based on version 9, using FlyBase release April 2015 (13). Based on this homology information a mutual crosslinking with FlyBase was established where the iBeetle-Base is included in the ‘Linkouts’ section (14). Up to now 6518 *Drosophila* genes are linked to 5853 *Tribolium* genes by 9774 homology relations. After implementation of the links, FlyBase has developed to one of the top referring pages for iBeetle-Base, only best by links from the genome browser of the iBeetle project. Such a crosslinking facilitates an easy comparison of phenotypes and the underlying gene function across different species and helps to overcome species specific characteristics.

**Table 1.** The statistics table shows the increase of the main numbers between the previous and the current release

What	Release 2015	Current	Increase
Number of TC genes	16 505 (OGS2)	16593 (OGS3)	—
Genes analysed by RNAi	5180	8665	67%
Phenotypic annotations	39 156	58 881	50%
Images documenting phenotypes	14 487	26 683	84%

In order to provide an accurate source for the selection of RNAi targets the genome was newly assembled based on additional sequencing data (15) (GCA_000002335.3) and the gene models enhanced by adding extensive RNA-seq data and a more accurate AUGUSTUS prediction (16) (OGS3). The new official gene set was incorporated in iBeetle-Base and all dsRNAs have been matched to the new gene model.

### Query pipeline as alternative way to retrieve data

**Figure 1.**
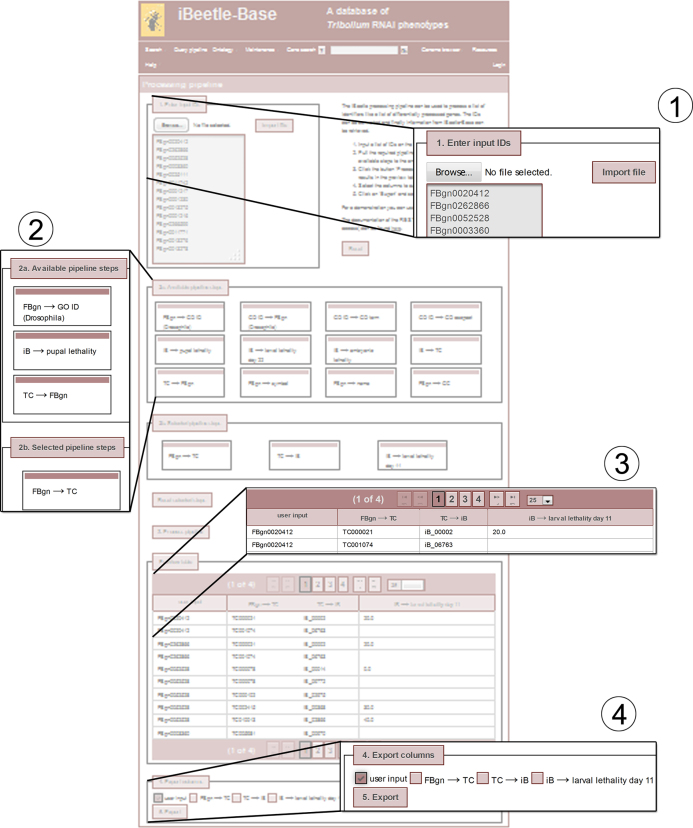
The figure shows a screenshot of the webpage for the query pipeline. The complete page is shown as transparent structure in the background. Important parts are enlarged. The workflow and the numbering of the cut-outs are: Step 1: Input a list of identifiers, or upload a text file with identifiers with one ID per row. Step 2 a/b: Drag and drop the required pipeline steps from ‘2a. Available pipeline steps’ to ‘2b. Selected pipeline steps’. Step 3: A click on the Button ‘3. Process pipeline’ executes the pipeline and presents the data in the preview table. Step 4: The preview table shows the output of each selected pipeline step. Step 5 Selected columns are exported to a csv file.

The main search interface of iBeetle-Base is the search for morphological defects. With the support of the morphological ontology for *Tribolium* TrOn and the ontology service OBA (ontology based answers) (17,18). This search is optimized to find genes relevant for the development of an anatomical structure. As alternative search, the quick search in the menu bar facilitates to retrieve information about a given gene. However, the retrieval of information for a list of genes required the manual identification of the respective gene IDs and subsequent navigation to the gene information pages. Therefore, a new query pipeline was implemented. The intended use of this pipeline is to start with a list of identifiers that can be converted into a list of related identifiers ultimately allowing to extract information from iBeetle-Base. A typical input for the pipeline is a list of genes from *Tribolium* or *Drosophila*, a list of iBeetle IDs obtained from a morphological search or GO terms (see step 1 in Figure 1 and on the webpage). Then, different converting steps can be selected and combined from a list of pipeline steps by drag and drop (step 2 a/b on Figure 1 and on the webpage). For instance, a list of differentially expressed genes from *Drosophila* can be converted into a list of *Tribolium* orthologs (TC numbers) and then into corresponding iBeetle IDs. These provide access to RNAi phenotypes at iBeetle-Base for comparison. The lethality values are good indicator for essential genes.

Importantly, the provided pipeline steps each perform one single conversion. This way, the pipeline is kept multifunctional and allows different ways of usage. Further the user stays aware of the performed steps, e.g. if GO terms, assigned to *Drosophila* genes are mapped to *Tribolium* genes.

After the pipeline has been executed the results are previewed in a table (step 4 on the Figure 1 and on the webpage) and selected columns can be exported to a csv file. In case of a one-to-many mapping the lines are duplicated, so that each cell always contains only a single value or each row a single dataset to facilitate further processing.

The input of a given step does not need to come from the immediately preceding step. Rather, the entire previous processing steps are searched for a matching input. This way, the input or any intermediate step can be used multiple times as input for another step.

The architecture of the query pipeline is designed to be simple and multifunctional. Each pipeline step is implemented as an endpoint of a RESTful interface (Representational State Transfer) emitting the data in JSON format (JavaScript Object Notification) (19). The design allows that each endpoint accepts a single ID or a comma separated list of IDs. The output is a map with the ID as key and the result as value, either as list or single value. IDs from the input that do not match the required pattern are ignored; IDs that have a valid format but do not exist or produce no output are copied as key to the result map with an empty value. The following example shows a HTTP GET request to the endpoint TC2FBgn, which retrieves homologous genes.

GET http://ibeetle-base.uni-goettingen.de/ibp/idmapper/TC2FBgn/ TC000085,TC009911

{‘TC009911’:[‘FBgn0020617’, ‘FBgn0000061’, ‘FBgn0008636’, ‘FBgn0085369’, ‘FBgn0023489’, ‘FBgn0003145’],

‘TC000085’:[‘FBgn0013799’]}

The documentation of the single endpoints is provided in JSON format. The documentation includes fields like the regular expression to validate the input (key ‘Input pattern’ in the documentation map). It is possible to include automatically any of the endpoints in a system, based on the documentation in JSON format. In iBeetle-Base all pipeline steps are registered and used without any code specific for the single steps. Below the documentation for the above used endpoint is given. (The keys of the JSON map are printed in bold for better readability.)

GET http://ibeetle-base.uni-goettingen.de/ibp/idmapper/TC2FBgn

{‘**Input**’:‘A singe TC ID or a comma separated list of TC IDs. Please note, that the length of the URL is limited by client, server and proxies.’,

‘**Description**’:‘The orthologous and homologous genes were calculated by OrthoDB. The list of homologous genes is limit to 6, orthologous and homologes genes are returned in the same list.’,

‘**Titel**’:‘TC → FBgn’,

‘**Output**’:‘The output is a map with the TC IDs from the input and a list of FBgn IDs for this TC ID ’,

‘**Example**’:[‘/ibp/idmapper/TC2FBgn/TC000085’, ‘/ibp/idmapper/TC2FBgn/TC000085,TC009911’],

‘**Exceptions**’:‘If the iB ID from the input is valid, but no data is found, the iB ID is copied to output with an empty value. If the iB ID has not a valid format, this ID is skipped.’,

‘**Summary**’:‘Get the ortholog and homolog Drosophila genes for a Tribolium gene.’,

‘**Input pattern**’:‘TC[0–9]{6}’}

### Proposing of GO terms by the community

Gene Ontology (GO) annotations are an import tool to define the function of a gene in a machine-readable manner (20). These terms are defined in very few highly-developed model organisms. However, based on orthology of a gene, GO terms can be retrieved for genes of other species as well (21,22). GO enrichment analyses provide valuable pieces of information for the formulation of hypotheses that guide subsequent work. Especially genomic approaches often lead to long lists of involved genes and GO term analysis has become a standard to get information on the character of the respective subset of genes. However, in the light of evolution where gene functions are expected to change over time, the focus on very few model systems might lead to a bias especially with respect to the biological function of genes. Therefore, it is essential that gene functions are studied in other organisms and that the respective results are made available to the community in form of GO terms. So far, results from *Tribolium* have not been added.

With the current release of iBeetle-Base we invite the community to provide GO terms of the genes they have expert knowledge of. On the details page for each Tribolium gene a subpanel named ‘Gene Ontology’ was added. Here, the GO terms of the homologous fly genes are listed on the right as reference. On the left, *Tribolium* specific GO terms will be listed. Below of that, a form can be opened to propose a new GO term. The mandatory fields are the TC gene (pre-populated), the GO term to assign to this gene, the evidence level (how the function of the gene was determined) and the literature reference which documents the statement. Not mandatory, but highly encouraged is to copy the relevant section of the reference into the quotation field. This information will later be displayed to the user and also helps the iBeetle-Base curators to verify the proposal, before committing it to the Gene Ontology consortium.

## DISCUSSION

Within insects, FlyBase is the only resource providing genome-wide information on gene function. With iBeetle-Base we develop the first alternative resource based on data gained in the emerging model organism *T. castaneum*. With the current version, we reached coverage of half of the genome while the analysis of the remaining genes is already under way. Hence, two genome-wide resources will be available in the near future for insects allowing the systematic comparison of gene functions across species. In order to facilitate such comparisons, respective tools and links need to be implemented. For single gene comparisons, the links to the respective pages of the other species are sufficient. For the processing of gene lists gathered in one species for analysis in both, the query pipeline was designed. Importantly, the architecture was designed for extensibility. Hence, upon internal or external requirements, more pipeline steps will be added.

The core of the phenotypic information of iBeetle-Base has been gathered by the iBeetle RNAi screen. However, numerous groups have been using the model system and a lot of information is available in the literature. It will be difficult for a single group to add all this information to the database. However, community contributions can help to increase the data. As first step to include data from the community we invite the researches to propose GO terms for the genes they are experts of. We selected GO terms as first type of community data because data analysis using GO terms has become an important tool in genomics with the drawback that such analysis is performed using homologous genes from very few model organisms.

## AVAILABILITY

iBeetle-Base is available at http://ibeetle-base.uni-goettingen.de. The RESTful interface for the pipeline is accessible at http://ibeetle-base.uni-goettingen.de/ibp
